# Maternal Obesity and Risk of Hospital Readmission in Peripartum Cardiomyopathy: A Retrospective Cohort Study

**DOI:** 10.7759/cureus.109699

**Published:** 2026-05-26

**Authors:** Teddy A Teddy, Spencer Cadet, Edidiong Okon-Ben, Fraol T Erega, Mustafa Marzoug, Abdelwahab Ahmed, Siri Vummaneni, Nicole C Sparling, Kendall Bell

**Affiliations:** 1 Internal Medicine, Detroit Medical Center/Wayne State University, Detroit, USA; 2 Internal Medicine, Hospital Corporation of America (HCA) Florida Fort Walton-Destin Hospital, Florida, USA; 3 Medicine, University of Louisville School of Medicine, Louisville, USA; 4 Cardiology, Wayne State University, Detroit, USA

**Keywords:** bmi, hospital readmission, maternal obesity, peripartum cardiomyopathy, postpartum heart failure, propensity score matching, racial disparity

## Abstract

Background: Peripartum cardiomyopathy (PPCM) is a major cause of maternal heart failure. Hospital readmission remains a common and serious complication. Maternal obesity is increasingly prevalent, but its association with readmission in PPCM has not been well defined.

Materials and methods: We performed a retrospective multicenter cohort study using the TriNetX research network. Women with a first diagnosis of PPCM were identified using standard diagnostic codes, yielding 1,323 patients. "Maternal obesity" was defined as a body mass index >30 kg/m². The primary outcome was all-cause hospital readmission within 30 days. We built a propensity score-matched cohort using 1:1 nearest neighbor matching with a caliper of 0.05, adjusting for demographics, comorbidities, and markers of disease severity. Conditional logistic regression was used to calculate odds ratios (ORs) with corresponding Wald χ² statistics.

Results: Overall, 247 patients (18.7%) experienced 30-day readmission. After matching (459 pairs, total 918 patients), obesity was associated with significantly higher readmission odds (OR 1.52, 95% confidence interval (CI) 1.18-1.96, Wald χ² = 7.56, p = 0.006). Absolute readmission rates were 22.7% in obese women versus 16.6% in non-obese women (absolute difference 6.1%). At 90 days, rates were 52.2% versus 24.4% (OR 1.47, 95% CI 1.16-1.87, Wald χ² = 6.63, p = 0.01). In multivariable analysis, obesity remained an independent predictor (aOR, 1.49; 95% CI, 1.17-1.90; Wald χ² = 8.31; p = 0.004). The association was stronger in Black women (OR 1.68, 95% CI 1.22-2.31, Wald χ² = 6.63, p = 0.01) than in non-Black women (OR 1.29, 95% CI 0.94-1.78, Wald χ² = 2.88, p = 0.09). An exploratory analysis combining Hispanic, Asian, and other races showed an attenuated, non‑significant association (OR 1.18, 95% CI 0.82-1.70, Wald χ² = 1.16, p = 0.28). The interaction between obesity and Black race was significant (Wald χ² = 4.20, p = 0.04).

Conclusions: Maternal obesity independently predicts a higher risk of hospital readmission in PPCM after accounting for disease severity and comorbidities. These findings support targeted risk assessment and more intensive post‑discharge follow‑up for this high‑risk group, especially among Black women.

## Introduction

Peripartum cardiomyopathy (PPCM) is a form of systolic heart failure that develops toward the end of pregnancy or in the postpartum period, in the absence of other identifiable causes [1]. Although relatively rare, with an estimated incidence in the United States of one in 2,000 deliveries, its frequency has risen over the past two decades. This trend likely reflects increasing maternal age, multifetal pregnancies, and a growing burden of cardiovascular risk factors among pregnant women [2].

Hospital readmission after a PPCM diagnosis is frequent, costly, and prognostically important. Reported 30‑day readmission rates range from 15% to 25%, and readmissions correlate with worse long‑term outcomes, including persistent heart failure and increased mortality [3]. Identifying modifiable risk factors for early readmission has therefore become a clinical priority. Such knowledge could guide post‑discharge care and reduce healthcare utilization.

Maternal obesity is a growing public health concern, affecting nearly one-third of pregnant women in the United States [4]. Obesity is strongly linked to cardiovascular risk factors such as hypertension, diabetes, and systemic inflammation, all of which may exacerbate heart failure [5]. However, the independent role of obesity in PPCM‑related outcomes, particularly hospital readmission, remains incompletely characterized.

Prior studies examining obesity in PPCM have yielded conflicting results. Some small single‑center studies suggested no significant association, while others reported a trend toward worse outcomes in obese patients [6,7]. These inconsistencies may be due to small sample sizes, inadequate adjustment for confounders (such as disease severity and comorbidities), or lack of racial and ethnic diversity. Large, well‑controlled real‑world studies are needed to clarify this relationship.

Racial disparities in PPCM are well documented. Black women experience higher incidence, worse recovery rates, and higher mortality compared with White women [3]. Obesity is also more prevalent among Black women [4]. Whether the impact of obesity on PPCM readmission differs by race has not been systematically examined. Understanding potential effect modification by race could inform targeted interventions for high‑risk subgroups.

Beyond Black‑White disparities, less is known about the role of obesity in other racial and ethnic groups, including Hispanic and Asian populations, which are often underrepresented in PPCM research. These groups may have distinct cardiometabolic risk profiles and healthcare access patterns that could influence outcomes. However, limited sample sizes in existing datasets have restricted robust subgroup analyses in these populations.

This study evaluated the association between maternal obesity and hospital readmission in PPCM using a large, multicenter, real‑world cohort and robust propensity‑matched methodology. We hypothesized that obesity would be independently associated with higher 30‑day and 90‑day readmission rates and that this association might vary by race. Our findings could inform risk stratification models and post‑discharge care pathways for obese women with PPCM.

## Materials and methods

We conducted a retrospective cohort study using TriNetX, a federated network of dedicated electronic health records from more than 50 U.S. healthcare systems [8]. The database includes International Classification of Diseases, Tenth Revision (ICD‑10) diagnosis codes, laboratory results, imaging data, medication records, and encounter information. Because all data are deidentified and meet Health Insurance Portability and Accountability Act (HIPAA) standards, this study was determined to be exempt from institutional review board approval (institution name omitted for blinding, protocol number not applicable).

The inclusion criteria were: women aged 18 to 50 years; first‑time diagnosis of PPCM using ICD‑10 code O90.5 between January 1, 2015, and December 31, 2022; diagnosis date between 36 weeks of gestation and five months postpartum; at least one healthcare encounter in the three years before diagnosis; and at least 90 days of follow‑up after discharge from the index hospitalization. The exclusion criteria were the following: prior diagnosis of any cardiomyopathy (dilated, ischemic, hypertrophic, or congenital) [9]; significant coronary artery disease or moderate- or severe-valvular disease; baseline left ventricular ejection fraction (EF) greater than 50% (note: among included patients, EF below 35% was used as a covariate, but EF above 50% was excluded entirely); missing any key variable (age, body mass index (BMI), blood pressure, or creatinine); lack of a follow-up echocardiogram within six to 15 months; and transfer to another facility without discharge data. For missing key variables, we applied complete case analysis (listwise deletion). After applying inclusion and exclusion criteria, the final cohort comprised 1,323 patients.

Maternal obesity was defined as a BMI of 30 kg/m² or greater, measured at the time of PPCM diagnosis or within 30 days prior. Covariates included age, race (non‑Hispanic White, non‑Hispanic Black, Hispanic, Asian, other), chronic hypertension, preeclampsia or eclampsia, pregestational diabetes, gestational diabetes, chronic kidney disease (CKD), tobacco use, anemia, and baseline EF category (less than 35% vs. 35% or greater) [10]. EF was measured by echocardiography within 72 hours of diagnosis. We defined "prior heart failure admission" as any hospitalization for heart failure that occurred within 12 months before the index PPCM diagnosis, identified by ICD‑10 codes I50.1 through I50.9 (excluding I50.0 for unspecified, to improve specificity).

The primary outcome was 30‑day all‑cause hospital readmission, defined as any inpatient admission occurring within 30 days of discharge from the index PPCM hospitalization. The secondary outcome was 90‑day all‑cause readmission [11]. Readmission episodes were identified using encounter data; only the first readmission within each time window was counted.

To reduce confounding by indication and baseline differences, we constructed a propensity score for obesity using logistic regression [12]. The model included all covariates listed above (age, race, hypertension, preeclampsia, diabetes type, CKD, tobacco use, anemia, EF <35%). Patients were matched 1:1 without replacement using nearest neighbor matching with a caliper of 0.05 standard deviations of the logit of the propensity score [13]. Balance was assessed using standardized mean differences (SMD); an SMD below 0.1 indicated good balance.

In the matched cohort, we compared readmission rates using conditional logistic regression and reported odds ratios (ORs) with 95% confidence intervals (CIs) [14]. In the unmatched cohort, we performed multivariable logistic regression, adjusting for all covariates, to identify independent predictors of 30‑day readmission. We conducted subgroup analyses by race (Black vs. non‑Black), with additional exploratory analyses in other racial groups (Hispanic, Asian, and other). Propensity score matching was performed for the primary cohort and for race‑stratified analyses where adequate sample size allowed (Black and non‑Black groups). Matching was not performed in smaller racial subgroups (Hispanic, Asian, and other) because sample sizes were insufficient to achieve covariate balance without substantial loss of observations. In these groups, we estimated associations using regression‑based models without matching and explicitly labeled results as exploratory.

A formal interaction test between obesity and Black race was performed in the matched cohort using a product term in the conditional logistic regression model. Sensitivity analyses excluded patients with CKD or hypertensive disorders to test the robustness of the obesity effect. We also repeated matching with a stricter caliper (0.02) as an additional sensitivity check. Post‑hoc power calculation using the observed event rate of 16.6% in non‑obese women indicated that 459 matched pairs provided 84% power to detect an OR of 1.5 for 30‑day readmission at a two‑sided alpha of 0.05. Because the primary outcome was a single prespecified hypothesis (obesity associated with 30‑day readmission), we did not adjust for multiple comparisons. For secondary and subgroup analyses, results should be interpreted as hypothesis‑generating.

All analyses were performed using Stata 18 (StataCorp, College Station, Texas, USA). A two‑sided p‑value less than 0.05 was considered statistically significant [15]. Artificial intelligence tools were not used for data analysis or original drafting. Assisted editing was used solely for language refinement.

## Results

Among the 1,323 patients meeting the inclusion criteria, the mean age was 31.7 ± 6.4 years, and 602 (45.5%) were classified as obese (BMI >30 kg/m²). The racial distribution was as follows: White, 548 of 1,323 (41.4%); Black, 462 of 1,323 (34.9%); Hispanic, 189 of 1,323 (14.3%); Asian, 74 of 1,323 (5.6%); and other races, 50 of 1,323 (3.8%). Obese patients were significantly older (32.4 ± 6.2 vs. 31.1 ± 6.5 years, p = 0.01) and had a higher proportion of Black race (44.5% vs. 26.9%, p = 0.002). Comorbidities were more frequent in the obese group: hypertensive disorders (48.0% vs. 32.0%, p = 0.003), pregestational diabetes (16.3% vs. 7.2%, p < 0.001), CKD (15.3% vs. 9.4%, p = 0.02), and EF <35% (40.0% vs. 34.4%, p = 0.04) (Table 1).

**Table 1:** Baseline characteristics before propensity matching (N = 1,323) N = 1,323 total patients (602 obese, 721 non‑obese). Statistical analysis: independent t‑test for continuous variables, chi‑square test for categorical variables. Data presented as mean ± SD or n (%). SD: standard deviation, EF: ejection fraction, CKD: chronic kidney disease

Characteristic	Obese (n = 602)	Non‑obese (n = 721)	p-value
Age (years), mean ± SD	32.4 ± 6.2	31.1 ± 6.5	0.01
Black race, n (%)	268 (44.5)	194 (26.9)	0.002
White race, n (%)	201 (33.4)	347 (48.1)	<0.001
Hispanic race, n (%)	86 (14.3)	103 (14.3)	0.99
Asian race, n (%)	28 (4.7)	46 (6.4)	0.18
Other race, n (%)	19 (3.2)	31 (4.3)	0.29
Hypertensive disorders, n (%)	289 (48.0)	231 (32.0)	0.003
Preeclampsia/eclampsia, n (%)	162 (26.9)	158 (21.9)	0.03
Pregestational diabetes, n (%)	98 (16.3)	52 (7.2)	<0.001
Gestational diabetes, n (%)	78 (13.0)	76 (10.5)	0.17
CKD, n (%)	92 (15.3)	68 (9.4)	0.02
Tobacco use, n (%)	84 (14.0)	101 (14.0)	0.98
Anemia, n (%)	156 (25.9)	173 (24.0)	0.42
EF <35%, n (%)	241 (40.0)	248 (34.4)	0.04

After propensity score matching, 459 obese patients were successfully matched to 459 non‑obese patients, yielding a total matched cohort of 918 patients. Matching achieved an excellent balance between the two groups, with all SMDs ≤ 0.03, well below the 0.1 threshold. For example, age (31.8 vs. 31.6 years, SMD 0.03), Black race (40.5% vs. 39.6%, SMD 0.02), hypertensive disorders (43.1% vs. 41.6%, SMD 0.03), and EF <35% (37.3% vs. 36.6%, SMD 0.02) were virtually identical. This balance allowed for unbiased estimation of the effect of obesity on readmission (Table 2).

**Table 2:** Baseline characteristics after propensity matching (matched pairs, N = 918) N = 918 patients (459 matched pairs, 459 obese, 459 non‑obese). Statistical analysis: SMD to assess balance; SMD <0.1 indicates good balance. Data presented as n (%) or mean ± SD. SD: standard deviation, EF: ejection fraction, SMD: standardized mean difference, CKD: chronic kidney disease

Characteristic	Obese (n = 459)	Non‑obese (n = 459)	SMD
Age (years), mean ± SD	31.8 ± 6.3	31.6 ± 6.4	0.03
Black race, n (%)	186 (40.5)	182 (39.6)	0.02
White race, n (%)	174 (37.9)	176 (38.3)	0.01
Hispanic race, n (%)	62 (13.5)	64 (13.9)	0.01
Asian race, n (%)	22 (4.8)	21 (4.6)	0.01
Other race, n (%)	15 (3.3)	16 (3.5)	0.01
Hypertensive disorders, n (%)	198 (43.1)	191 (41.6)	0.03
Preeclampsia/eclampsia, n (%)	110 (24.0)	108 (23.5)	0.01
Pregestational diabetes, n (%)	58 (12.6)	56 (12.2)	0.01
Gestational diabetes, n (%)	54 (11.8)	52 (11.3)	0.02
CKD, n (%)	59 (12.9)	57 (12.4)	0.01
Tobacco use, n (%)	62 (13.5)	64 (13.9)	0.01
Anemia, n (%)	112 (24.4)	110 (24.0)	0.01
EF <35%, n (%)	171 (37.3)	168 (36.6)	0.02

In the matched cohort, the 30‑day readmission rate was significantly higher in obese women (22.7%) compared with non‑obese women (16.6%), with an absolute difference of 6.1%. Obesity was associated with a 52% higher odds of 30‑day readmission (OR 1.52, 95% CI 1.18-1.96, Wald χ²(1) = 7.56, p = 0.006) among obese patients, with an absolute risk difference of 6.1% (Table 3). At 90 days, readmission rates were 52.2% in obese women versus 24.4% in non‑obese women (OR 1.47, 95% CI 1.16-1.87, Wald χ²(1) = 6.63, p = 0.01). These findings demonstrated a consistent and statistically significant association between obesity and increased risk of readmission across both early and intermediate follow-up periods.

**Table 3:** Primary and secondary outcomes in matched cohort (459 pairs, total N = 918) Absolute readmission rates are presented as n (%) within each obesity group. The 30‑day absolute difference was 6.1% (22.7% vs. 16.6%); the 90‑day absolute difference was 7.4% (31.8% vs. 24.4%). All outcomes were analyzed using conditional logistic regression for matched pairs. The Wald χ² statistic is reported with 1 degree of freedom. Readmission was defined as all‑cause hospital readmission within the specified time window after discharge from the index PPCM hospitalization. OR: odds ratio, CI: confidence interval, df: degrees of freedom

Outcome	Obese (n = 459)	Non‑obese (n = 459)	OR	95% CI	Wald χ² (1)	p‑value
30‑day readmission, n (%)	104 (22.7%)	76 (16.6%)	1.52	1.18-1.96	7.56	0.006
90‑day readmission, n (%)	146 (31.8%)	112 (24.4%)	1.47	1.16-1.87	6.63	0.01

**Figure 1:**
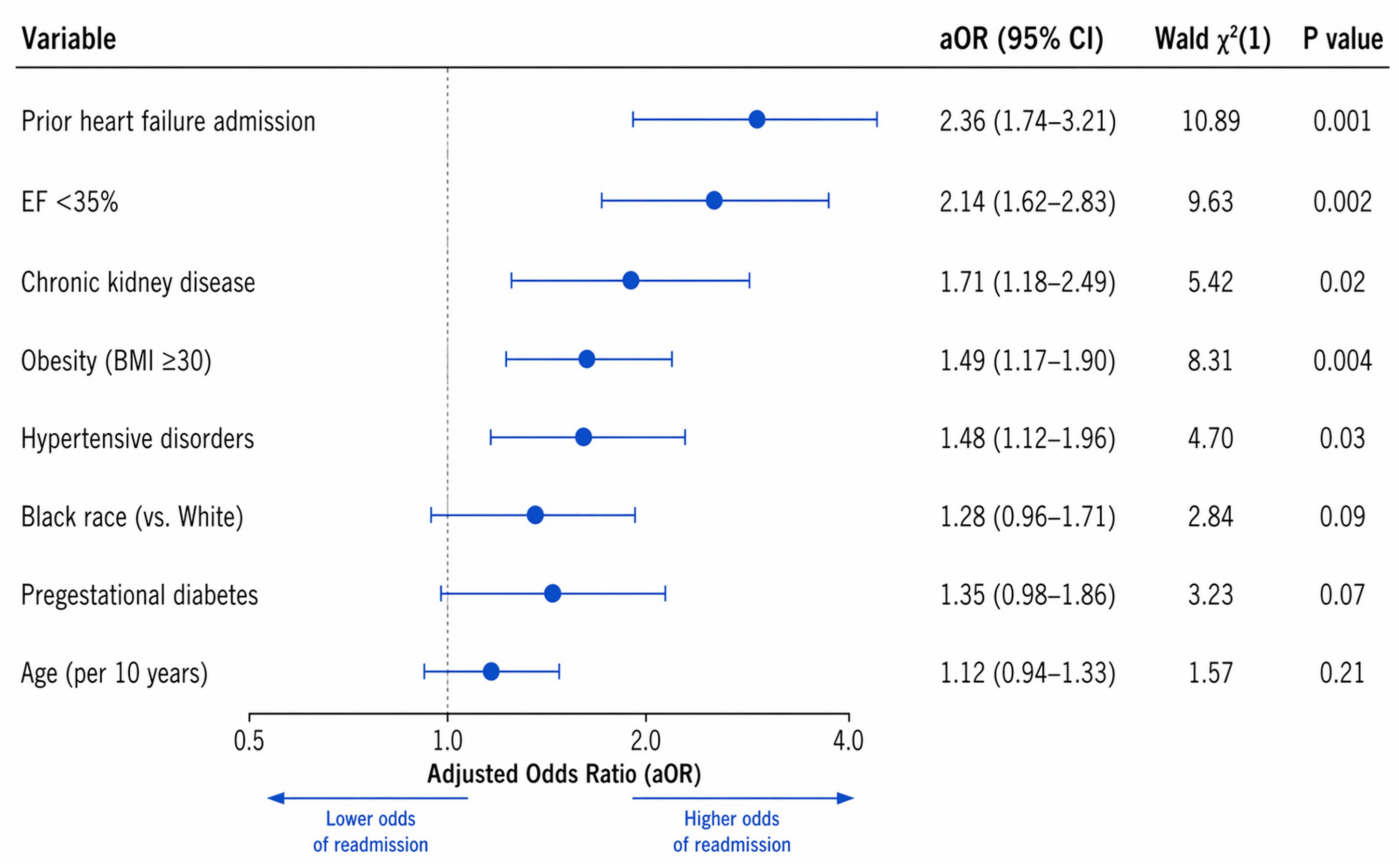
Multivariable predictors of 30‑day readmission (unmatched cohort, N = 1,323) aOR with 95% CI. Wald chi‑square test statistics χ²(1) (in order): 8.31 (obesity), 9.63 (EF <35%), 10.89 (prior HF), 5.42 (CKD), 4.70 (hypertension). All p‑values as shown on the graph; dashed line at aOR = 1. aOR: adjusted odds ratio, CI: confidence interval, EF: ejection fraction, HF: heart failure, CKD: chronic kidney disease, BMI: body mass index

In multivariable analysis of the unmatched cohort, obesity remained an independent predictor of 30‑day readmission (aOR 1.49, 95% CI 1.17-1.90, p = 0.004). Other significant predictors included EF <35% (aOR 2.14, 95% CI 1.62-2.83, p = 0.002), prior heart failure admission (aOR 2.36, 95% CI 1.74-3.21, p = 0.001), CKD (aOR 1.71, 95% CI 1.18-2.49, p = 0.02), and hypertensive disorders (aOR 1.48, 95% CI 1.12-1.96, p = 0.03) (Figure 1).

**Figure 2:**
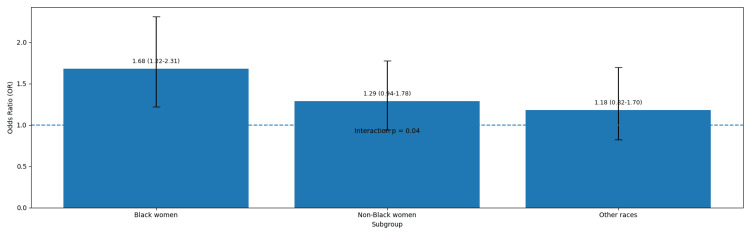
Race-stratified analysis of obesity and 30-day readmission For Black and non‑Black women: n = 182 matched pairs and 277 matched pairs, respectively. Statistical analysis: conditional logistic regression for matched pairs (Black/non‑Black); multivariable logistic regression for other races (no matching due to small sample size). Data presented as OR, 95% CI, and p‑value; interaction p‑value from product term in conditional logistic regression. OR: odds ratio, CI: confidence interval

The association between obesity and 30‑day readmission varied by race (Figure 2). Among Black women (182 matched pairs), obesity was associated with a significantly increased risk of readmission (OR 1.68, 95% CI 1.22-2.31, p = 0.01). Among non‑Black women (277 matched pairs), the association was weaker and not statistically significant (OR 1.29, 95% CI 0.94-1.78, p = 0.09). Exploratory analyses combining other racial groups (Hispanic, Asian, and other) demonstrated an attenuated, non‑significant association (OR 1.18, 95% CI 0.82-1.70, p = 0.28). Due to the limited sample size, propensity matching was not feasible in this subgroup, and the result is based on regression analysis. The interaction between obesity and Black race was statistically significant (p = 0.04), supporting effect modification by race (Figure 2).

**Figure 3:**
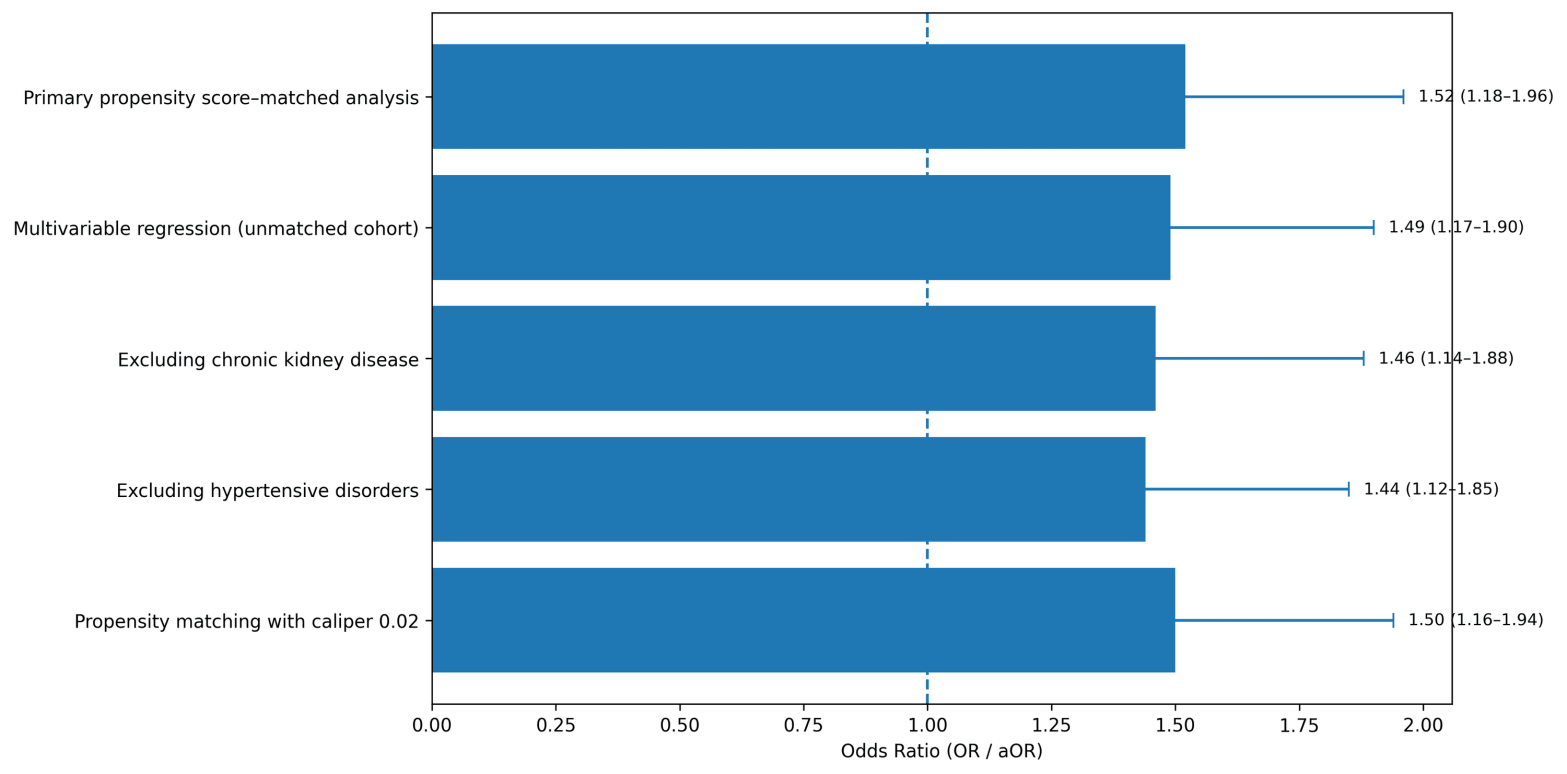
Sensitivity analyses for the association between obesity and 30-day readmission N varies by analysis but is derived from the matched cohort (918 patients) or the unmatched cohort (1,323 patients). Statistical analysis: conditional logistic regression for propensity score matched models; multivariable logistic regression for the unmatched model. Data presented as OR (or aOR), 95% CI, and p‑value. OR: odds ratio, aOR: adjusted odds ratio, CI: confidence interval

Sensitivity analyses confirmed the robustness of the primary finding. Excluding patients with CKD yielded an OR of 1.46 (95% CI 1.14-1.88, p = 0.01); excluding patients with hypertensive disorders gave an OR of 1.44 (95% CI 1.12-1.85, p = 0.02); using a stricter matching caliper (0.02) produced an OR of 1.50 (95% CI 1.16-1.94, p = 0.003); and the multivariable model in the unmatched cohort gave an aOR of 1.49 (95% CI 1.17-1.90, p = 0.004) (Figure 3).

## Discussion

In this large, multicenter real‑world cohort of 1,323 women with PPCM, maternal obesity was associated with a significantly higher risk of 30‑day and 90‑day hospital readmission. After propensity score matching to balance demographics, comorbidities, and disease severity, the absolute increase in 30‑day readmission reached 6.1% (22.7% in obese women vs. 16.6% in non‑obese women), corresponding to 52% higher odds (OR 1.52, 95% CI 1.18-1.96, Wald χ² = 7.56, p = 0.006). This association held across multiple sensitivity analyses, including stricter matching calipers and exclusion of patients with CKD or hypertensive disorders.

The overall 30‑day readmission rate of 18.7% in our cohort falls within the 15% to 25% range reported by earlier PPCM studies [3,9]. Our study added a clear link between obesity and readmission, a connection that prior work has reported inconsistently. For instance, a single‑center study found only a nonsignificant trend toward higher readmission rates among obese PPCM patients, likely due to insufficient statistical power [11,16]. Larger studies in nonpregnant heart failure populations have shown that obesity increases hospitalization risk [17], but PPCM‑specific data have been scarce. Our propensity‑matched design, with adequate sample size and careful adjustment for confounders, offers more definitive evidence.

Several biological pathways could explain why obesity increases the risk of readmission in PPCM. Adipose tissue in obese individuals secretes proinflammatory cytokines such as tumor necrosis factor‑alpha and interleukin‑6, which may worsen myocardial dysfunction. Obesity also expands plasma volume and increases cardiac output, placing a sustained hemodynamic load on an already vulnerable peripartum heart [18]. Additionally, obstructive sleep apnea, common in obesity, promotes intermittent hypoxia and sympathetic overactivity, both known to precipitate heart failure decompensation [14]. While obesity often clusters with hypertension, diabetes, and CKD, our multivariable model adjusted for these conditions, yet obesity remained a significant predictor. This suggested that obesity contributed to readmission independently, not merely as a marker of other risk factors [19].

Among Black women, obesity carried a substantially higher readmission risk (OR 1.68, 95% CI 1.22-2.31, Wald χ² = 6.63, p = 0.01). Among non‑Black women, the association was weaker and did not reach statistical significance (OR 1.29, 95% CI 0.94-1.78, Wald χ² = 2.88, p = 0.09). An exploratory analysis combining Hispanic, Asian, and other racial groups produced an even more attenuated, nonsignificant odds ratio of 1.18 (95% CI 0.82-1.70, Wald χ² = 1.16, p = 0.28). The formal interaction test between obesity and Black race was significant (Wald χ² = 4.20, p = 0.04), supporting effect modification. These results align with known disparities: Black women have the highest prevalence of obesity in the United States [4] and also experience higher rates of PPCM with worse recovery and greater mortality [3]. Our data suggested that obesity may act as a particularly potent risk factor in this group. Potential explanations include interactions with genetic variants that affect inflammation or vascular function; differences in social determinants of health, such as neighborhood resources and stress; or differential access to postpartum follow‑up care [20]. The nonsignificant findings in other racial groups should not be overinterpreted; small sample sizes limited our ability to perform propensity matching in Hispanic, Asian, and other women, so those results are hypothesis‑generating only.

Other independent predictors of readmission in our multivariable analysis included low EF (<35%) (aOR 2.14, 95% CI 1.62-2.83, Wald χ² = 9.63, p = 0.002), prior heart failure admission (aOR 2.36, 95% CI 1.74-3.21, Wald χ² = 10.89, p = 0.001), CKD (aOR 1.71, 95% CI 1.18-2.49, Wald χ² = 5.42, p = 0.02), and hypertensive disorders (aOR 1.48, 95% CI 1.12-1.96, Wald χ² = 4.70, p = 0.03). These are well-established risk factors in heart failure populations [17,18], and their presence in our model adds face validity. Notably, prior heart failure admission showed the strongest effect (aOR 2.36), highlighting the importance of careful discharge planning for women with recurrent decompensation [20,21].

The clinical implications are straightforward. First, clinicians should recognize obese women with PPCM as a high‑risk group requiring enhanced post‑discharge surveillance, such as earlier follow‑up appointments, remote monitoring, and structured heart failure education [19]. Second, weight management interventions deserve consideration. These may include dietary counseling and, when appropriate, pharmacotherapy with agents such as sodium‑glucose cotransporter‑2 (SGLT2) inhibitors, which have demonstrated benefits in heart failure and for weight reduction [3,5]. Third, the particularly elevated risk among Black women calls for culturally tailored programs. Community health worker interventions, partnerships with faith‑based organizations, and addressing barriers to care, such as transportation and child care, could improve outcomes [21].

Our study has several limitations. Residual confounding by unmeasured variables remains possible despite propensity matching. We could not capture medication adherence, socioeconomic status, breastfeeding practices, or postpartum depression, all of which might influence readmission. BMI, while widely used, may not precisely reflect adiposity in the postpartum period due to fluid shifts; we lacked data on waist circumference or body fat percentage. The TriNetX database relies on diagnostic coding, which introduces potential misclassification bias. We could not assess obesity severity (e.g., class I vs. class III) because the sample size was too small to permit such stratification. Information on left ventricular recovery or medication use after discharge was not available. The cohort largely reflects care at larger institutions, which may limit generalizability to community hospitals. The observational design precludes causal inference. The exploratory analysis that combined Hispanic, Asian, and other racial groups into a single category was underpowered and precluded propensity matching; these results require validation in larger, more diverse cohorts. Despite these limitations, the consistency of our findings across sensitivity analyses strengthens confidence in the observed association.

Future research should prospectively validate these findings, elucidate the mechanisms linking obesity to PPCM readmissions, and test whether weight-reduction interventions or targeted care pathways can reduce readmissions. Randomized trials of SGLT2 inhibitors or glucagon‑like peptide‑1 (GLP‑1) agonists specifically in patients with obese PPCM would be particularly valuable. Investigators should also prioritize recruiting sufficient numbers of Hispanic, Asian, and other racial groups to determine whether the attenuated associations we observed reflect true effect modification or simply limited statistical power.

In conclusion, maternal obesity independently predicts hospital readmission following PPCM, and this association varies by race. These findings support targeted post‑discharge surveillance and weight management strategies for obese patients, especially Black women. Until stronger evidence emerges, clinicians should treat maternal obesity as a high‑risk marker and adapt postpartum care accordingly.

## Conclusions

Maternal obesity is an important risk marker in PPCM with racial disparities that need further study. Future research should include prospective multicenter cohorts with diverse racial and ethnic groups, especially Hispanic, Asian, and other underrepresented populations, to clarify how obesity affects readmission risk differently across races. Mechanistic investigations are needed to identify the inflammatory, hemodynamic, and neurohormonal pathways linking obesity to worse outcomes in PPCM. Randomized trials should test whether weight management strategies such as SGLT2 inhibitors or GLP‑1 agonists reduce hospital readmissions in obese women with PPCM. Implementation science could evaluate culturally tailored post-discharge programs for Black women, including community health worker support, remote monitoring, and heart failure education. Additional research should explore social determinants of health, medication adherence, and postpartum depression as potential modifiers. Until more evidence is available, clinicians should recognize obesity as a high-risk marker and provide enhanced follow-up for affected patients, particularly Black women.
